# Unmet needs for modern contraceptive methods among sexually active adolescents and young women in Togo: a nationwide cross-sectional study

**DOI:** 10.3389/fpubh.2023.1169180

**Published:** 2023-07-27

**Authors:** Smaila Alidou, Lucien Désiré Dahourou, Ter Tiero Elias Dah, Armel Sogo, Tchasso Serge Kenao, Dègninou Yehadji, Nicolas Meda, Didier Koumavi Ekouevi

**Affiliations:** ^1^Département de Santé Publique, Unité de Formation et de Recherche en Sciences de la Santé, Université Joseph KI-ZERBO, Ouagadougou, Burkina Faso; ^2^Ministère de la Santé, Hygiène Publique et de l’Accès Universel aux Soins, Lomé, Togo; ^3^Institut de Recherche en Sciences de la Santé (IRSS/CNRST), Ouagadougou, Burkina Faso; ^4^Institut National de la Santé et de la Recherche Médicale (INSERM, UMR1295), Centre d’épidémiologie et de Recherche en Santé des Populations (CERPOP), Université de Toulouse, Toulouse, France; ^5^Unité de Formation et de Recherche en Sciences de la Santé, Université de Ouahigouya, Ouahigouya, Burkina Faso; ^6^Département de Promotion de la Santé, Institut Régionale de Santé Publique, Université d’Abomey-Calavi, Cotonou, Benin; ^7^Département de Santé Publique, Faculté des Sciences de la Santé (FSS), Université de Lomé, Lomé, Togo

**Keywords:** modern contraceptives, unmet need, adolescent and young women, household factors, Togo

## Abstract

**Background:**

The unmet need for modern contraceptives among sexually active adolescent and young women (AYW) in Africa contributes to high morbidity and mortality. To investigate the prevalence of unmet need for modern contraceptives and its associated factors among AYW in Togo, we performed a secondary analysis of data from the MICS-62017 survey.

**Method:**

We extracted data from sexually active AYW aged 15–24  years for the analysis and used multi-level logistic regression models to identify factors associated with unmet need for modern contraceptives.

**Results:**

Among the AYW, the median age was 20  years. The prevalence of unmet need for modern contraceptives was 27.02%. Factors that increased the likelihood of having unmet need for contraceptives included being in the “Poor” or “Middle” quintile of household wealth, aged 20–24  years, and completing primary or secondary education. Living in a household headed by a woman and having a household head aged 19–38, 39–58, or greater than 78  years decreased the likelihood of unmet need for modern contraceptives.

**Conclusion:**

The study highlights the high-unmet need for modern contraceptives among sexually active AYW in Togo and emphasizes the importance of addressing individual and household/community factors to improve their sexual and reproductive health. Interventions such as increasing AYW awareness, providing social marketing campaigns in schools, and targeting men-headed households could help promote modern contraceptive use and improve the sexual and reproductive health of AYW in Togo.

## Introduction

The sexual health of adolescents and young people and their access to modern contraceptive methods, is an underlying principle of the Sustainable Development Goals (SDGs), including SDG3 to enable all people to live in good health and promote well-being for all at all ages (1, 2) and is a fundamental component of the Global Strategy for Women’s, Children’s and Youth Health (2016–2030) (3). According to the World Health Organization (WHO), adolescents are people aged 10–19 years and young people aged 15–24 years (4). In 2019, youth and adolescents were estimated to number about 1.3 billion worldwide, with the majority living in developing countries, particularly in sub-Saharan Africa (SSA) (5) and it is projected that by 2050, youth and adolescents living in SSA will be estimated at around 850 million (6).

The majority of young people in SSA are exposed to a number of challenges, growing up under the weight of customs and traditions, in an environment of insecurity coupled with high unemployment and limited educational opportunities and access to health services (7, 8). Apart from these common challenges, young adolescent girls are at high risk of reproductive health problems, gender-based violence, unwanted pregnancies and unsafe abortion (5, 9). Each year, approximately 74 million unintended pregnancies occur worldwide, the majority of which are among young girls. Ninety-five per cent of the 16 million births each year to girls aged 15–19 are reported in developing countries (DCs) (10, 11).

In SSA, complications related to pregnancy, childbirth and unsafe abortion are the leading causes of death for young girls (12). In 2019, complications related to pregnancy and childbirth accounted for the deaths of 27,000 adolescent girls (13). Unintended and early pregnancies not only compromise the physical, psychological and socio-economic well-being of young people and adolescents, but also constitute a significant psychological and physical risk factor for children born from these pregnancies (14).

Contraceptive use has been globally recognized as essential to control fertility (15, 16). It is a major component of women’s reproductive health, particularly for young adolescents who have a desire to avoid, space or limit childbirth (17). Although contraceptives have been considered effective in regulating fertility, their use among young adolescents in DCs, particularly in SSA, remains an issue that requires urgent attention (13, 18). Studies have shown that, despite the desire of young girls and older women in SSA to use contraceptives, the majority of them face difficulties accessing contraceptive services (19, 20). A useful measure for of the gap between the desire for and access to contraception is the estimation of unmet need for contraception (21, 22).

According to the World Health Organization (WHO), women with unmet need for contraception are those who are fertile and sexually active but not using any method of contraception, and who report that they do not want another child or want to delay the next one (23). This is an important indicator not only because it provides essential information for monitoring the achievement of specific goal 3.7.1 of the Sustainable Development Goals (SDGs) (2) but also because it shows the extent to which a country is fulfilling the reproductive health rights of its population (17). In 2019, it was estimated that approximately 270 million women had an unmet need for contraceptives worldwide, with great disparity between regions of the world, countries and individuals (24–27). Indeed, about 30% of young women under the age of 25 have an unmet need for modern contraceptives in SSA (26, 28). Darroch et al. estimated that if all unmet need for modern contraception were met among adolescents, there would be 59% fewer unintended pregnancies, 67% fewer unplanned births and 57% fewer induced abortions per year, and 71% fewer maternal deaths (29).

In Togo, according to the 2017 Multiple Indicator Cluster Survey (MICS-6), young adolescent girls represent 39.1% of the population (30). As in other SSA countries, they are a key population to benefit from sexual and reproductive health (SRH) interventions, including modern contraceptives (5). Over the past decade, Togo has created an enabling environment for adolescent SRH, including a legal framework and national plans (31–33). However, little is known about the unmet need for modern contraceptives among young Togolese adolescents. Using data collected in the 2017 MICS survey, this analysis aimed to estimate the prevalence of unmet need for modern contraception, and to identify its associated factors among young adolescent girls aged 15–24 in Togo.

## Methods

### Study design and population

We conducted a secondary analysis of data from the MICS-6 conducted in Togo in 2017. For the present analysis, we included adolescent girls and young women aged between 15 and 24 years, sexually active (i.e., indicated their first sexual intercourse age), and giving the response to the question about the use of contraceptive.

### Sampling and data collection

MICS is developed and funded by The United Nations Children’s Fund (UNICEF). It is a national population-based survey for supporting countries in the collection of wide range and representative indicators in maternal and child health, including unmet needs for modern contraceptive (30).

The study population was selected using a two-stage stratified sampling. The primary sampling units (PSUs) included clusters. They were selected using the sampling frame of the 2010 General Census of Population and Housing (RGPH4) of Togo (34). A total of 420 PSUs (253 in rural settings, and 167 in urban settings) were selected for the survey. The household were the secondary sampling units. Twenty of them were selected in each PSU, giving 8,400 households. All the women living in the household or who stayed there last night, and aged between 15 and 49 years old, were eligible and invited to participate in the survey (3). A total of 7,326 women participated in the MICS 2017.

A large range of data including sociodemographic, as well as sexual and reproductive health information were collected using a standardized questionnaire administered by trained interviewers.

### Study variables

#### Dependent variables

Unmet need for modern contraceptives (for spacing or limiting births) was the dependent variable. It was operationalized into two categories (Yes/No). Married and unmarried, fertile and sexually active AYW aged 15–24 years who wished to avoid, space or limit pregnancies but were not using a modern contraceptive method were considered to have an unmet need for modern contraceptives (26, 35, 36), AYW who had never had sex and those who were infecund/menopausal were excluded.

The concept of unmet need for modern contraception is a statistical construct and its equation can be written as follows:


$$\begin{array}{l} \mathrm{Unmet need for moderns contraceptive}=\\ \frac{\left(\mathrm{Modern contraceptive need}-\mathrm{Modern contraceptive}\;\mathrm{use}\right)\;}{\left(\mathrm{Modern contraceptive need}+\mathrm{Non}-\mathrm{use}\;\mathrm{of modern methods}\right)} \end{array}$$

#### Independent variables

Potential explanatory variables were selected *a priori* on the basis of existing literature about unmet needs for modern contraceptives (28, 36–38). They included individual variables, and others related to household or community.

The individual level variables included age, marital status, education level, media exposure and parity. Age was coded as “15–19” and “20–24.” Marital status was recoded into “Single,” and “in couple.” Level of education was coded as “no education,” “primary,” and “secondary and above.” Media exposure was coded as “Not at all,” “Less than once a week,” and “At least once a week.” Parity was recoded as “No children,” “[1–2],” and “[3 and up].”

The household or community level variables were wealth quintile, age of household head, sex of household head, education level of household head, place of residence, region of residence and household size. The wealth quintile was generated using principal component analysis (PCA) (30, 39) and was classified as poorest, poor, middle, richer and richest. Age of household head was coded as “≤18,” “[19–38],” “[39–58],” “[59–78],” and “>78.” Sex of household head was coded as “male” and “female.” Education level of household head was coded as “no education,” “primary,” and “secondary and above.” Place of residence was coded as “urban” and “rural.” Region of residence was coded as “Grand-Lomé,” “Maritime,” “Plateaux,” “Central,” “Kara,” and “Savanes.” Household size was code as “[1–3],” “[4–6],” and “[7 and up].”

### Statistical analysis

The analyses were performed taking into account the sample weight. Qualitative variables were described by their frequency with 95% confidence intervals (CI) and quantitative variables were described by their median and interquartile range (IQR: Q3–Q1). To investigate the association between unmet need for contraception and the explanatory variables, we used the chi-square [χ2] test of independence.

To identify factors associated with unmet needs for modern contraceptives, we used a multi-level logistic regression model (MLRM) with fixed and random-effects. A two-level model for binary responses was developed. At the first level, unmet needs for contraception were reported to the AYW. At the second level, it was reported to the household/community. Four regression models were used. The first “empty model/null model,” which shows the variance in unmet need for modern contraceptives, attributed to clusters primary sampling units (PSUs). This model has no explanatory variables. The second model included only the individual variables. The third model included only the household or community variables. The fourth model included all the variables controlling for both individual and household/community variables. A collinearity test (variance inflation factor) was used to check the correlation between the explanatory variables.

The MRLMs are mixed models with fixed and random effects (40, 41). Fixed effects measured the association between the explanatory variables and the dependent variable, estimated as an adjusted Odd Ratio (aOR) with their 95% confidence interval (95%CIs). Random effects are measures of variation, estimated using the Intra-class Correlation Coefficients (ICC) (41). The ICC is the proportion of the variation in unmet need for modern contraceptives explained by the cluster effect (PSU). The higher the CCI, the more the clusters (PSUs) influence the variation in unmet need for modern contraceptives (41, 42). This means that the CCI should be taken into account when interpreting the results of the logistic regression models. The likelihood of having an unmet need for modern contraceptives may vary depending on which PSU the respondent belongs to Li et al. and Goldstein (43, 44). Therefore, the CCI may affect the estimates of the aORs and their significance levels (45).

To evaluate the model adequacy, we used the Likelihood Ratio test. We also used Akaike’s Information Criterion (AIC) and Bayesian Information Criteria (BIC) to measure how well the different models fitted the data.

All statistical analyses were performed using STATA software version 16.0. We set the statistical significance level at *p* < 5% for all analyses.

### Ethical considerations

The MICS data are owned by UNICEF. The MICS-62017 was the fifth session held in Togo and was approved by the National Bioethics Advisory Committee in July 2017. We obtained the authorization for using the data after an electronic request on the survey’s website (http://mics.unicef.org/surveys).

## Results

**Figure 1:**
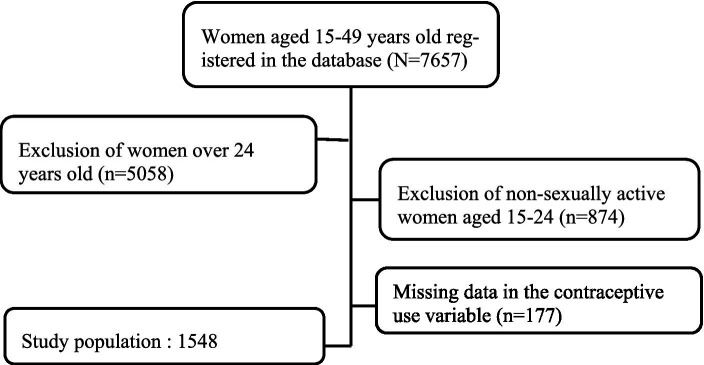
Data inclusion flow chart, MICS-62017, Togo.

A total of 7,657 women aged between 15 and 49 years were included in the MICS 2017 survey. Of these, 1,548 women were included in the present analysis (Figure 1).

The median age of the AYG was 20 years (IQR: 22–18 years). The majority of AYW had a secondary education (57.81%) level, were single (54.93%) and were nulliparous (50.61%). They belonged mostly to households located in urban areas (82.92%), headed by men (66.87%) and households from the rich quintile (24.95%).

The prevalence of unmet need for modern contraceptives among sexually active girls in Togo was 27.02% [95%CI: 24.87–29.29]. It was high among young adolescents aged 20–24 years (28.31%), those with secondary education and above (32.2%), unmarried girls (30.0%), those exposed to the media at least once a week (31.00) and nulliparous girls (30.10). There were also variations in the prevalence of unmet need for modern contraceptives by household/community characteristics. Young adolescents from households headed by heads aged 59–78 years (30.00%), those from male-headed households (29.40%), living in rural areas (27.70%), in the Maritime region (30.80%) and Grand Lomé region (30.06), and those from households in the very rich quintile (32.40%). The results of the Chi-square test indicate that with the exception of girls’ age and parity as individual characteristics, and the level of education of the head of household, place of residence, area of residence, and household size as household/community factors, all other variables are significantly associated with unmet need for modern contraceptives (Table 1).

**Table 1:** Distribution of unmet need for modern contraceptives among sexually active adolescents and young girls in Togo according to explanatory variables, MICS 2017 (Weighted).

Variables	Number (*n*)	Proportion (%)	Unmet need for modern contraceptives		*p*-value
Individual levels variables			Yes	No	
Age group (*n* = 1,548)					
[15–19]	591	40.2	25.1	74.9	
[20–24]	957	59.8	28.3	71.7	
Marital status (*n* = 1,548)					0.009
Single	853	54.9	30.0	70.0	
In couple	695	45.1	23.4	76.6	
Level of education (*n* = 1,548)					0.000
No education	210	13.3	11.9	88.1	
Primary	416	28.9	23.6	76.4	
Secondary and above	922	57.8	32.2	67.8	
Media exposure (*n* = 1,548)					0.002
Not at all	405	25.2	18.2	81.8	
Less than once a week	214	14.2	25.4	74.6	
At least once a week	929	60.6	31.0	69.0	
Parity (*n* = 1,548)					0.085
No children	788	50.6	30.1	69.9	
[1–2]	673	44.1	23.7	76.3	
[3 and up]	87	5.3	25.4	74.6	
Household/community levels					
Age of household head (*n* = 1,425)					0.023
≤18	9	0.7	74.0	26.0	
[19–38]	448	32.7	29.0	71.0	
[39–58]	605	43.9	24.6	75.4	
[59–78]	299	18.6	30	70	
>78	64	4.1	24.6	75.4	
Gender of household head (*n* = 1,425)					0.035
Female	479	66.9	23.4	76.6	
Male	946	33.1	29.4	70.6	
Education level of household head (*n* = 1,425)					0.798
No education	474	31	26.4	73.6	
Primary	476	32.4	28.4	71.6	
Secondary and above	475	36.6	28.4	71.6	
Place of residence (*n* = 1,546)	1,546				0.376
Rural	298	82.9	27.7	72.3	
Urban	1,248	17.1	23.8	76.2	
Region of residence (*n* = 1,548)					0.136
Grand Lomé	436	28.6	30.6	69.4	
Maritime	142	11.3	30.8	69.2	
Plateaux	252	27.7	27.0	73.0	
Centrale	246	10	22.1	77.9	
Kara	221	11.6	27.9	72.1	
Savanes	251	10.8	17.1	82.9	
Well-being index (*n* = 1,548)					0.003
Poorest	239	14.1	15.9	84.1	
Poor	290	17.9	21.4	78.6	
Middle	345	20.8	29.1	70.9	
Richer	321	22.3	30.6	69.4	
Richest	353	24.9	32.4	67.6	
Household size (*n* = 1,425)					0.604
[1–3]	644	44.3	25.7	74.3	
[4–6]	580	40.8	28.7	71.3	
[7 and up]	201	14.9	28.8	71.2	

### Fixed effects (measure of association)

The Model 3 (Table 2) is the comprehensive model that presents the association between individual and household/community factors and unmet need for modern contraceptives among young girls. For individual factors, AYG age and education were associated with unmet need for modern contraceptives. Household/community factors such as age of head of household, gender of head of household and household wealth quintile index were significantly associated with unmet need for modern contraceptives.

**Table 2:** Multivariate analysis of factors associated with unmet need for modern contraceptives among girls aged 15–24 in Togo, MICS 2017 survey.

Variables	OR [IC95%]			
	Model 0	Model 1	Model 2	Model 3
Fixed effects results				
Individual level variables				
Age of the girls				
[15–19]		1		1
[20–24]		1.53 [1.14–2.05]^**^		1.56 [1.14–2.15]^**^
Marital status				
In couple		1		1
Single		1.15 [0.79–1.69]		1.14 [0.75–1.72]
Educational level young people				
No education		1		1
Primary		2.13 [1.24–3.65]^**^		1.94 [1.10–3.40]^*^
Secondary and above		2.91 [1.70–4.96]^***^		2.61 [1.48–4.60]^***^
Media exposure				
Not at all		1		1
Less than once a week		1.27 [0.81–2.00]		1.18 [0.72–1.92]
At least once a week		1.46 [1.02–2.09]^*^		1.22 [0.81–1.84]
Parity				
No children		1		
[1–2]		0.85 [0.85–1.25]		0.82 [0.54–1.24]
[3 et plus]		1.35 [0.67–2.73]		1.25 [0.60–2.62]
Household/community levels variables				
Age of head of household				
≤18			1	1
[19–38]			0.13 [0.03–0.64]^*^	0.16 [0.03–0.84]^*^
[39–58]			0.10 [0.02–0.50]^**^	0.13 [0.03–0.65]^*^
[59–78]			0.16 [0.03–0.80]^*^	0.19 [0.04–0.99]^*^
>78			0.12 [0.02–0.68]^*^	0.16 [0.03–0.92]^*^
Gender of head of household				
Male			1	1
Female			0.68 [0.51–0.92]^*^	0.66 [0.49–0.90]^**^
Region of residence				
Maritime			1	1
Plateaux			1.03 [0.57–1.87]	1.06 [0.58–1.91]
Centrale			0.67[0.34–1.35]	0.69 [0.35–1.37]
Kara			0.93 [0.47–1. 82]	0.98 [0.50–1.92]
Savanes			0.56 [0.27–1.16]	0.66 [0.32–1.36]
Lomé commune			0.71 [0.40–1.27]	0.69 [0.39–1.23]
Well-being index				
Poorest			1	1
Poor			1.65 [0.96–2.85]	1.43 [0.82–2.48]
Middle			2.51 [1.43–4.39]^**^	2.01 [1.13–3.58]^*^
Riche			2.44 [1.35–4.41]^**^	1.77 [0.93–3.34]
Richest			2.95 [1.61–5.39]^***^	2.03 [1.06–3.84]^*^
Random effects results				
PSU variance (CI: 95%)	0.89 (0.56–1.40)	0.68 (0.40–1.15)	0.72 (0.42–1.24)	0.64 (0.36–1.16)
ICC (SE)	0.21	0.17	0.18	0.16
LR test	X2 = 58.62^***^	X2 = 35.85^***^	X2 = 35.94^***^	X2 = 27.96^***^
Wald X2	Reference	37.75^***^	39.14^***^	60.40^***^
Model fitness				
log-likelihood	−875.9	−855.7	−783.9	−771
AIC	1755.7	1731.1	1599.9	1,590
BIC	1766.4	1752.9	1684.04	1,675
Number of cluster	1,548	1,548	1,548	1,548

Exponentiated coefficients; 95% confidence intervals in brackets; AOR, adjusted Odds Ratios; CI, Confidence Interval. ^*^p < 0.05; ^**^p < 0.01; ^***^p < 0.001; ICC, Intra-class correlation; SE, Standard Error; PSU, Primary Sampling Unit; LR Test, Likelihood ratio Test; AIC, Akaike’s Information Criterion; BIC, Schwarz’s Bayesian. Model 0 is the null model, a baseline model without any determinant variable. Model 1 is adjusted for individual level variables. Model 2 is adjusted for household/community level variables. Model 3 is the final model adjusted for individual and household/community level variables.

In regard with individual factors, the likelihood of unmet need for modern contraceptives was significantly higher among girls aged 20–24 years (aOR =1.54; [95%95CI: 1.13–2.10]) compared with those aged 19–20 years; those with a primary school (aOR = 1.90; [95CI: 1.09–3.35]) and those with secondary school education or higher (aOR = 2.56; [95%CI: 1.46–4.84]) compared with those with no education.

With regard to household/community factors, compared to AYW from the poorest quintile households, the likelihood of unmet need was significantly higher for AYW from middle households (aOR = 2.15; [95%CI: 1.20–3.84]) and the richest households (aOR = 2.03; [95%CI: 1.07–4.85]). In contrast, the likelihood of unmet need was significantly lower for AYW whose households head were female (aOR = 0.67; [95%CI: 0.49–0.90]), whose heads of households aged 19–38 (aOR = 0.17; [95%CI: 0.03–0.84]), 39–58 (aOR = 0.13; [95%CI: 0.03–0.67]) and over 78 (aOR = 0.16; [95%CI: 0.08–0.95]), compared to their female counterparts whose households were headed by men and household heads under the age of 18 (Table 2).

### For measures of variations (random effects)

The empty model reports that there is marginal variation in the probability of unmet need for modern contraceptives among girls in Togo that is explained by the clustering of Primary Sampling Units (σ2 = 0.89, 95% CI 0.56–1.40).

The null model shows that 21% of the total variance in unmet needs for contraception was attributed to variation between cluster characteristics (ICC = 0.21). The variation between clusters decreases by 4% in model 1, from 21% in the empty model to 17% in the individual-level model only. From Model 1, the ICC increased by 1% (ICC = 0.18) in Model 2 at the community level to decrease to 16% in the full model (Model 3), which contains both individual and household/community level variables. This demonstrates that variations in the probability of unmet need for modern contraceptives could be attributed to differences in clustering in the Primary Sample Units (PSUs).

The AIC and BIC values show a successive reduction which shows an improvement in each of the models compared to the previous one, thus affirming the goodness of fit of the final model. Therefore, the full model, composed of individual and household/community factors, was retained to identify factors associated with unmet need for modern contraceptives among girls aged 15–24 in Togo.

## Discussion

This nationwide representative study shows that more than one quarter (27.02%) of sexually active adolescent girls and young women aged 15–24 in Togo had an unmet need for modern contraceptives. Unmet need was significantly associated with age, education level of girls and age of household head, sex of household head and wealth quintile.

The prevalence of unmet need for modern contraceptives among adolescents and young people aged 15–24 reported in the present study was higher than that reported among adolescents and young people in eight out of 10 high-fertility countries in SSA (28) but lower than that reported among the adolescent and young girl population in Benin, Ghana, Cote d’Ivoire and the average unmet need in West and Central Africa reported by MacQuerrie (46). The high prevalence reported in our study compared to those reported among young women in other countries could be explained by the fact that in traditionally natalist environments, several obstacles inhibit young girls’ contraceptive intention. These include societal norms, financial and geographic accessibility, legal or illegal restrictions imposed by some health workers, and misconceptions about the fertility side effects of modern contraceptives (13, 47). This disparity could also be due to differences in location, study population and study period. Intensified interventions to increase the supply of and demand for contraceptive methods are needed to reduce this prevalence and the risk of unwanted pregnancies with their adverse consequences among adolescents and young girls. Also, high unmet needs were observed in the Maritime region and Grand Lomé, which could be attributed to the positive discrimination policy of free distribution of contraceptives in disadvantaged communities. This policy was implemented in Togo, particularly in the Savanes, Kara, Central, and part of the Plateaux region (48, 49).

The likelihood of unmet need for modern contraceptives was higher among young women aged 20–24 years, compared with their counterparts aged 15–19 years. These results differ from those reported in previous studies in SSA (26, 28, 37, 50). In other studies of women of childbearing age, it has been reported that girls aged 15–24 years are likely to have an unmet need for modern contraceptives (37, 51). The high likelihood of unmet need for modern contraceptives among 20–24 year old reported in this study could be explained by the strong influence of cultural values and societal norms on fertility desire and need for modern contraceptives (52, 53). In our settings, women generally adopt contraception only after a first birth (13, 21, 52, 54). This result also points to an issue of women’s empowerment. Indeed, the majority of women aged 20 to 24 in our settings are already in union (55). In these unions, they are under strong societal pressure to conceive a first child soon after the union (56). Thus, contraception is only considered after the birth of children desired by the husband or other members of the in-laws without taking into account the women’s opinion. The empowerment of women and the reduction of gender inequalities is therefore essential to reduce the unmet need for modern contraceptives in our context.

Girls with primary school and girls with secondary education and above had a high probability of unmet need for modern contraceptives compared to those with no education. Similar findings have been reported in other studies among girls in SSA (26, 28), Nigeria (42) and Ghana (37). As Solanke et al. (42) and Ahinkorah (28) reported in their studies, this result was counterintuitive. However, one possible reason could be that girls with higher levels of education have more information about potential side effects, which may reflect low use of modern contraceptives. The same remarks were made by Guure et al. (37). These results deserve to be further investigated through qualitative studies. It is also important to communicate better about the risk–benefit of using contraceptive methods in order to counter rumors and misinformation and to reassure those who have doubts about the safety of these methods.

In relation to household/community factors, girls from female-headed households were found to have a lower likelihood of having unmet needs for modern contraceptives compared to their male-headed counterparts. This finding is different from those reported in other studies conducted in Sub-Saharan Africa (26, 28), but similar to those reported in Benin (57) and in Tanzania (58). It is suggested that this finding could be related to the sexual health education provided by mothers to their young adolescent daughters for whom they are solely responsible. Such young people are more likely to benefit from their experience and information on responsible sexuality (59). which can have an impact on their decision-making regarding sexual health and contraceptive use (58, 60). Elsewhere, these results may also be explained by the presence of stigmatization, taboos, and societal norms that hinder men from addressing issues of sexual education with individuals under their care, particularly with girls (61, 62). Therefore, it is crucial to promote parental involvement in the sexual education of their children as a preventative measure against adverse health outcomes, unintended pregnancies, and premature deaths.

Regarding the age of household heads, the study also found that the probability of unmet need among young adolescent girls was lowest when they resided in households headed by householders over the age of 18, compared to those from households headed by householders aged 18 years or younger. These results are comparable to those reported in Uganda and Tanzania (58, 63). There could be several reasons to explain these results. Firstly, household heads over the age of 18 may have better knowledge and experience regarding reproductive health (64, 65), and additionally, they may have greater decision-making and can encourage the best decisions for sexual and reproductive health power (66, 67), which could facilitate access to family planning services for women in their households. On the other hand, younger household heads may face challenges in providing access to family planning services due to their own limited knowledge and experience, as well as societal norms and barriers (47, 52, 53) that limit their ability to make decisions related to reproductive health.

The study also reported a significant association between unmet need for modern contraceptives and the household wealth index. Girls in poor and in richest quintile were more likely to have an unmet need for modern contraceptives than those in the poorest quintile. These results are similar to those reported in Ghana by Guure et al. (37), but different from earlier studies in Nigeria (42) and several SSA countries (26, 28). This difference could be explained in part by the positive discrimination policy of free contraceptive products in favor of poorest and disadvantaged people in Togo (48, 49). Nevertheless, these results raise questions about how contraceptive issues are approached in the young women living in a favorable socio-economic household and deserve to be better investigated.

The main limitation of this study is the possibility of information bias related to social desirability. In fact, the AYW included in this study may provide acceptable responses to their views on contraceptive use and sexual behavior. Also, the use of secondary data limited our choice of explanatory variables. The use of nationally representative MICS data with a focus on sexually active young adolescents is a major strength of this study. Elsewhere, it should be noted that most of the factors identified in our study are not modifiable in the short term. The sampling procedures, the use of well-established procedures such as interviewer training and the use of validated MICS instruments reinforce the validity of the conclusions drawn from the data set.

## Conclusion

The study found that more than a quarter of sexually active young adolescents in Togo have an unmet need for modern contraceptives. Individual and household/community factors, including age and education level of young adolescents, age of head of household and socioeconomic status, were associated with unmet needs for modern contraceptives. We suggest that stakeholders involved in the sexual and reproductive health of AYW consider these factors in the development and implementation of interventions to promote the use of modern contraceptive methods. The improvement of AYW awareness, provision and social marketing campaigns in school, and a community-based communication targeting men-headed households could be helpful to improve the sexual and reproductive health of AYW adolescent girls in Togo.

## Data availability statement

Publicly available datasets were analyzed in this study. This data can be found at: http://mics.unicef.org/surveys.

## Ethics statement

The studies involving human participants were reviewed and approved by National Bioethics Advisory Committee of Togo. Written informed consent to participate in this study was provided by the participants’ legal guardian/next of kin.

## Author contributions

SA and LD: conceptualization, data curation, methodology, and resources. SA, LD, and AS: formal analysis. SA, DY, and AS: software. LD, TD, NM, and DE: supervision. LD, TD, DY, NM, and DE: validation. SA: writing—original draft preparation. LD, TD, DY, TK, DY, NM, and DE: writing—review and editing. All authors contributed to the article and approved the submitted version.

## Conflict of interest

The authors declare that the research was conducted in the absence of any commercial or financial relationships that could be construed as a potential conflict of interest.

## Publisher’s note

All claims expressed in this article are solely those of the authors and do not necessarily represent those of their affiliated organizations, or those of the publisher, the editors and the reviewers. Any product that may be evaluated in this article, or claim that may be made by its manufacturer, is not guaranteed or endorsed by the publisher.
